# Presence of chelonid fibropapilloma-associated herpesvirus in tumored and non-tumored green turtles, as detected by polymerase chain reaction, in endemic and non-endemic aggregations, Puerto Rico

**DOI:** 10.1186/2193-1801-1-35

**Published:** 2012-10-17

**Authors:** Annie Page-Karjian, Fernando Torres, Jian Zhang, Samuel Rivera, Carlos Diez, Phillip A Moore, Debra Moore, Corrie Brown

**Affiliations:** 1 Department of Pathology, University of Georgia, College of Veterinary Medicine, Athens, GA 30602 USA; 2 Foreign Animal Disease Diagnostic Laboratory, USDA-APHIS, Plum Island, Greenport, NY 11944 USA; 3 Zoo Atlanta, Atlanta, GA 30315 USA; 4 Puerto Rico Department of Natural and Environmental Resources, San Juan, 00906-6600 Puerto Rico; 5 Department of Small Animal Medicine and Surgery, University of Georgia, College of Veterinary Medicine, Athens, GA 30602 USA; 6 Caribbean Center of Marine Studies, Lajas, 00667-0585 Puerto Rico

**Keywords:** CFPHV, *Chelonia mydas*, Fibropapillomatosis, Herpesvirus, Laser capture microdissection, Nested PCR

## Abstract

Fibropapillomatosis (FP), a transmissible neoplastic disease of marine turtles characterized by a likely herpesviral primary etiology, has emerged as an important disease in green sea turtles (*Chelonia mydas*) over the past three decades. The objectives of this study were to determine the suitability of three different chelonid fibropapilloma-associated herpesvirus (CFPHV) gene targets in polymerase chain reaction (PCR) assays of affected tissues; to explore the presence of CFPHV in non-affected skin from turtles with and without tumors; and to better understand tissue localization of the CFPHV genome in a tumor-free turtle by evaluating CFPHV presence in microanatomic tissue sites. Two aggregations of green sea turtles (*Chelonia mydas*) in Puerto Rico were evaluated, with six sampling intervals over the three-year period 2004–2007. Primary and nested PCR for three different herpesviral gene targets- DNA polymerase, capsid maturation protease, and membrane glycoprotein B- were performed on 201 skin biopsies taken from 126 turtles with and without external tumors. Laser capture microdissection and nested PCR were used to identify tissue localizations of CFPHV in skin from a normal turtle. Of the turtles sampled in Manglar Bay, 30.5% had tumors; at the relatively more pristine Culebrita, 5.3% of turtles sampled had tumors. All three PCR primer combinations successfully amplified CFPHV from tumors, and from normal skin of both tumored and tumor-free turtles. Via nested PCR, the polymerase gene target proved superior to the other two gene targets in the positive detection of CFPHV DNA. CFPHV infection may be common relative to disease incidence, supporting the idea that extrinsic and/or host factors could play a transforming role in tumor expression. Laser capture microdissection revealed CFPHV in skin from a tumor-free turtle, harbored in both epidermal and dermal tissues. Identification of CFPHV harbored in a non-epidermal site (dermis) of a tumor-free turtle indicates that virus is latent in a non-tumored host.

## Background

Fibropapillomatosis (FP) is a transmissible neoplastic disease of marine turtles that is characterized by a variable number of cutaneous and conjunctival growths, with occasional appearance in visceral organs. The tumors often cause affected animals to become debilitated by hampering feeding and movement, obscuring vision, or in the case of visceral tumors, leading to organ failure (Balazs 1986 Herbst 1994; Jacobson et al. 1989; Quackenbush et al. 1998; Smith & Coates 1938). Over the past three decades, FP has emerged as an important disease in green sea turtles (*Chelonia mydas*), and is potentially threatening to their survival. Recent studies show that FP disease prevalence is now declining in Hawaiian green turtle populations; however FP prevalence in green turtles in Florida seems to be more stable (Chaloupka et al. 2009; Foley et al. 2005). Better understanding of the causation, pathogenesis, and diagnosis of marine turtle FP could help greatly in control efforts against this devastating disease.

The typical histologic description of FP includes papillary epidermal hyperplasia supported by broad fibrovascular stalks, with a varying ratio of epidermal to dermal proliferation (Herbst 1994). Papillomas are characterized by proliferating epidermis with little or no underlying dermal involvement; lesions composed predominantly of proliferating dermal components with a relatively normal overlying epidermis are characterized as fibromas. Fibropapillomas are thus classified as masses in which hyperplasia is observed in both epidermis and dermis (Herbst 1994). Visceral tumors are typically identified as fibromas, myxofibromas, or fibrosarcomas (Norton et al. 1990; Work et al. 2004). Visceral tumors tend to develop late in the course of disease, and are generally perceived as a more chronic lesion (Harshbarger 1991; Herbst 1994; Herbst et al. 1999; Jacobson et al. 1989; Lucke 1938).

The etiologic agent most often associated with FP is an alphaherpesvirus, designated chelonid fibropapilloma-associated herpesvirus (CFPHV). This virus has been partially sequenced but to date has resisted isolation attempts (Greenblatt et al. 2005; Herbst et al. 2004). The consistent association of this virus with FP tumors, either via polymerase chain reaction (PCR) or demonstrated through *in situ* hybridization, as well as effective disease transmissibility using cell-free tumor extracts, have resulted in a commonly perceived causal associative link (Herbst et al. 1995; Herbst et al. 2004; Kang et al. 2008; Lackovich et al. 1999; Lu et al. 2000; Quackenbush et al. 2001). However, the virus has been shown to be present, either serologically or through molecular techniques, in some normal animals. This raises the possibility that there are additional factors – ecologic, immunologic, or microbial – involved in disease development (Herbst et al. 2008; Lackovich et al. 1999; Lu et al. 2000; Quackenbush et al. 2001).

Identification of CFPHV deoxyribonucleic acid (DNA) in sea turtle tissues has been accomplished by various techniques, including PCR and *in situ* hybridization (Herbst et al. 1999; Kang et al. 2008; Lackovich et al. 1999; Lu et al. 2000; Quackenbush et al. 1998; Quackenbush et al. 2001). CFPHV DNA has been isolated from the following tissues: cutaneous tumors (i.e., fibropapillomas), tumors on the serosal surfaces of lungs, esophageal mucosa, and trachea (i.e. fibromas), scar tissue where an FP tumor was previously removed, and non-tumored tissues- skin, lungs, kidney, heart, spleen, liver, brain, periorbital tissue, conjunctiva, ovary, testis, tongue, gall bladder, intestine, urinary bladder, thyroid (Herbst et al. 1999; Jacobson et al. 1989; Kang et al. 2008; Lackovich et al. 1999; Lu et al. 2000; Quackenbush et al. 1998; Quackenbush et al. 2001).

The sloughing of virally infected epidermal cells from a diseased turtle into the surrounding environment is considered a source of viral shedding. Thus FP tumors are suggested to undergo viral shedding, as evidenced by the presence of CFPHV-associated intranuclear viral inclusions within cells of the epidermal strata germinativum and spinosum of some cutaneous tumors (Jacobson et al. 1989; Jacobson et al. 1991; Lackovich et al. 1999). In one study, the high prevalence of CFPHV glycoprotein H antibodies in a green turtle population with 0% tumor prevalence suggests that robust antibody responses to natural infection may develop independently of the appearance of cutaneous tumors (Herbst et al. 2008). Because CFPHV’s primary target seems to be skin, early or latent infection may be represented by the presence of CFPHV DNA in non-tumored skin from turtles with and without tumors (Quackenbush et al. 2001). There is one report of CFPHV polymerase DNA identified in differentiated epidermal and dermal tissues of tumors sampled from green turtles (Work et al. 2009). To date, however, there are no previous reports using the microanatomic location of CFPHV DNA to investigate potential viral shedding from the skin of “normal” turtles (i.e., turtles that do not have tumors). In the present study, application of an innovative technique, laser capture microdissection, permits a precise way to begin assessing the role that cell type plays in disease processes.

The study described here had three objectives. The first was to determine the suitability of various CFPHV gene targets in PCR assays for detection of CFPHV in affected tissues. The second objective was to explore the presence of CFPHV DNA in non-affected skin from both turtles with tumors and tumor-free turtles. The third objective was to use an innovative technology, laser capture microdissection, to evaluate CFPHV presence in epidermal and dermal tissues of normal skin sampled from a tumor-free turtle, in an effort to better identify the tissue location of the CFPHV genome.

## Results

Number of turtles sampled by location, including numbers of turtles with and without tumors, is presented in Table 1. Within six sampling sessions over a three year period, 126 turtles were captured and a total of 201 skin biopsy samples were taken.

**Table 1:** **Number of turtles sampled by location**, **with and without tumors**

Location	Total turtles	Turtles with tumors	Turtles without tumors
Manglar	69	18	51
Culebrita	57	3	54
TOTALS	126	21	105

Three different primer sets were designed for three CFPHV genes– DNA polymerase catalytic subunit (UL30, *pol*), capsid maturation protease (UL26), and membrane glycoprotein B (UL27, *gB*), and these nested CFPHV PCR targets were confirmed according to gene fragment size. The gene targets were further verified by the results of the Clustal^b^ multiple sequence alignments, which were conducted on the three nested PCR products- one sample of each type of nested amplicon. Alignment of the sequences revealed that the CFPHV DNA *pol* PCR product showed 100% (206/206bp) nucleotide sequence similarity; the capsid maturation protease PCR product showed 99.4% (322/324bp) nucleotide sequence similarity; and the membrane glycoprotein B PCR product showed 100% (300/300bp) nucleotide sequence similarity when compared to predicted homologous nucleotide sequences for Hawaiian green turtle herpesvirus, GenBank^a^ AF035003 (data not shown).

Results of the primary and nested PCRs for the three target sequences, i.e., DNA *pol*, capsid maturation protease, and membrane glycoprotein B, are shown in Table 2. Of the 38 tumor tissues, 89.5% were positive for the polymerase gene target by either primary or nested PCR. Primary PCR for the polymerase gene target was positive for six tumors, whereas nested PCR was positive for polymerase for all but five tumors (86.8%).

**Table 2:** Number of positive amplifications of target gene partial sequences^*^P = primary PCR; N = nested PCR

Description of Samples			DNA target type^*^					
			Polymerase		Capsid protease		Glycoprotein B	
Location	Type of tissue	Number	P	N	P	N	P	N
Manglar	Tumor	29	4	24	3	21	1	20
	Skin from turtle with tumor	18	0	8	0	9	0	5
	Skin from normal turtle	71	0	21	1	19	0	17
Culebrita	Tumor	9	2	9	0	2	2	9
	Skin from turtle with tumor	3	0	1	0	1	0	1
	Skin from normal turtle	71	0	13	1	7	1	9

Primary PCR for the capsid maturation protease gene target was positive in three tumors; nested PCR was positive for capsid maturation protease in 23 tumors. Primary PCR for the virion membrane glycoprotein B gene target was positive in three tumors; nested PCR was positive for glycoprotein B in 29 tumors.

In all but one case, when turtles with tumors were captured, skin from a non-tumored area was also collected. None of these 21 skin biopsies were positive by primary PCR, but 47.6% were positive for CFPHV using the nested PCR technique.

Additionally, skin samples were taken from 105 turtles that had no evidence of tumors, and assayed for presence of CFPHV. Of 142 skin biopsies taken from the 105 normal animals sampled, 32.4% were positive for nucleic acid of the virus by PCR. Of these, only 4.3% were positive using the primary PCR technique, and one of these was positive for both capsid maturation protease and membrane glycoprotein B. Of the 46 skin samples which tested positive by nested PCR, 39.1% were positive for both polymerase and capsid maturation protease. Three other normal skin samples were only positive via nested PCR for membrane glycoprotein B.

Using laser capture microdissection, DNA extraction, and the DNA *pol* target nested PCR protocol, CFPHV DNA was detected in both epidermal and dermal tissues from the skin of a non-tumored turtle. Distinct bands were visualized in the approximately 364bp region of the gel for both epidermal and dermal samples, consistent with the positive control for the CFPHV DNA polymerase gene target. The nested PCR product sequences representing the differentiated dermis and epidermis samples were subjected to Clustal^b^ multiple sequence alignment to verify the gene targets. Comparison of the dermis CFPHV DNA polymerase PCR product showed 96% (288/300bp) nucleotide sequence similarity; and comparison of the epidermis CFPHV DNA polymerase PCR product showed 98% (331/338bp) nucleotide sequence similarity when compared to predicted homologous nucleotide sequences for the Hawaiian green turtle herpesvirus DNA polymerase catalytic subunit gene *pol* (GenBank^a^ AF035003). The dermis CFPHV PCR product was noted to be relatively impure, which could explain the low sequence similarity obtained for this product as compared to the other sequences reported here.

## Discussion

Of the turtles sampled in Manglar Bay during these six sampling sessions, 30.5% had tumors. At the relatively more pristine Culebrita, 5.3% of turtles sampled had tumors. A Fisher’s exact test revealed a significant association between proportion of turtles with FP and location (Manglar Bay vs. Culebrita, p = 0.0005, α = 0.05). Historical records provided by the Puerto Rico Department of Natural Resources indicate that in past surveys tumors were common in turtles found in Manglar Bay but rare in turtles within the Culebrita aggregation. Specifically, turtles with FP were reported at Manglar Bay with high to medium prevalence, i.e. 57% in 2001–2005 and 30% in 2006–2007 (Diez et al. 2010). In more recent years, however, FP prevalence has been reported to be as low as 0% at Manglar Bay (Patricio et al. 2011). Sampling at Culebrita showed that FP was rare (<1% prevalence) until 2009, when a 40% FP disease prevalence was observed in green turtles captured there (Patricio et al. 2011; Velez-Zuazo et al. 2010).

In this study, the tumors were collected immediately into formalin and then after 12–24 hours, changed to RNase-free PBS. This was logistically necessary, due to the tropical working environment- the high ambient temperatures made it impossible to keep ice frozen for the duration of an entire sampling day. Although this technique worked even with brief formalin fixation, in future studies if freezing the tissues is not feasible a more appropriate technique will be employed to conserve the DNA, such as use of 98% ethanol or a ribonucleic acid (RNA) stabilization reagent.^c^

One aim of this study was to determine the suitability of various CFPHV gene targets in PCR assays for detection of CFPHV in affected tissues. To accomplish this, we tested three different gene targets for the CFPHV genome, and applied primary and nested PCR assays to detect the DNA in both tumored and non-tumored tissues. The PCR technique was successful at amplifying the virus from tumors, with polymerase nested PCR performing the most consistently. All but one of the 34 CFPHV-positive tumors yielded a positive result using the nested PCR technique for the polymerase gene target (97.2%). Our results are comparable to other studies which have described the use of nested PCR technique for polymerase gene target to detect CFPHV DNA in tumor tissues. The CFPHV DNA was detected in 100% and 95.7% of tumors examined from green turtles in Hawaii and Florida, respectively (Lackovich et al. 1999; Lu et al. 2000). The study reported here is an important addition to previous work because it compares three different gene targets for CFPHV DNA detection. As expected, the polymerase gene target proved to be superior to the capsid maturation protease and membrane glycoprotein B gene targets in the positive detection of CFPHV DNA. It is vital, however, not to overlook the utility of the alternative gene targets. The one tumor which was negative by nested PCR for polymerase was positive for CFPHV DNA by nested PCR for both capsid maturation protease and membrane glycoprotein B. So, the overall sensitivity of viral detection may be improved in future studies by the use of these additional targets.

Another aim of this study was to explore the presence of CFPHV DNA in non-tumored skin taken from turtles with and without tumors. In addressing this aim, we found that several normal skin samples taken from tumored turtles were CFPHV positive by PCR. Of 21 biopsies taken from normal shoulder skin in animals that had tumors at other sites, 47.6% were positive by nested PCR for the polymerase gene and occasionally the other two DNA targets. These results agree with those of previous reports documenting CFPHV DNA in normal skin from turtles with tumors. In two studies conducted in Hawaiian green turtles with tumors, 57.1% and 93.3% of skin samples were positive by nested PCR for the polymerase gene, respectively (Lu et al. 2000; Quackenbush et al. 1998). In normal skin sampled from tumored green turtles in Australia, 45.5% of samples were positive for the polymerase gene by quantitative PCR (Quackenbush et al. 2001).

The CFPHV DNA was also detected in skin from “normal” turtles, i.e., those without any apparent tumors. There were 105 normal turtles captured and biopsied, and 32.4% of 142 biopsy samples were positive for CFPHV. None of these were positive by primary PCR for the polymerase gene, whereas 34 were positive by the nested PCR technique. These results are similar to those reported from another part of the world. Of skin samples from 14 normal green turtles in Australia, 21.4% were positive for the CFPHV polymerase gene by quantitative PCR (Quackenbush et al. 2001). However, the findings in our study are also contradictory to some other published reports. In a study on non-tumored, stranded green turtles in Florida, all skin samples were negative by nested PCR for the CFPHV polymerase gene (Lackovich et al. 1999). In two studies conducted in Hawaiian green turtles, all skin samples from normal green turtles were negative by nested PCR (Lu et al. 2000; Quackenbush et al. 1998). The differences in these various studies are not easily explained. Some natural variations can be expected based on the widely different geographic regions, population genetics, and overall environmental considerations. But also, some of the differences in the reported works may be a function of sample size. The work presented here represents a significant contribution to the existing research because the sample size is considerably larger than any previously reported, and may be a more accurate representation of the true nature of the situation.

Separation of dermis and epidermis can be accomplished by gross dissection alone; however, dissection of tissues inherently could lead to sample contamination by unsolicited cell types. Laser capture microdissection allows for precise localization of a specific segment of nucleic acid within a histologic section. As such, this technique was applied to address the third aim of our study: to better identify the precise tissue location of the CFPHV genome in skin sampled from a non-tumored turtle. We employed laser capture microdissection to determine whether CFPHV DNA in a non-tumored section of skin might be harbored in epidermis or dermis, or both. We specifically chose to use skin from a “normal” turtle, and we used a biopsy that had previously been shown to contain CFPHV nucleic acid via PCR. Separating samples from epidermis and dermis yielded surprising results- the viral nucleic acid was present in both portions. A previous study documents CFPHV DNA in both dermis and epidermis of FP tumors, with higher levels in the dermis (Work et al. 2009). Because viral particles have routinely been observed in epidermal cells of tumored skin, it is generally assumed that tumored skin undergoes viral shedding, at least periodically, and that transmission may occur in this manner. But our findings are notable in that we also found viral nucleic acid in differentiated tissues of normal turtle skin, suggesting there may be viral shedding from non-tumored turtles as well. Additionally, the identification of viral nucleic acid in dermis as well as epidermis raises the possibility that the infection is much more than an epidermal infection, but that there may be systemic cells that also harbor the virus.

## Conclusions

The results of this study constitute a meaningful contribution to related evidence that CFPHV infection may be common relative to disease incidence, and support the idea that aspects of the environment and host may play a transforming role in FP disease expression. This idea is further supported by phylogenetic analyses of CFPHV which show evidence of low viral mutability, suggesting coevolution of the virus with marine turtle hosts over millennia and a potential for external factors to affect disease expression (Herbst et al. 2004; Patricio et al. 2012). It is known that turtles develop FP after they recruit to nearshore habitat locations as juveniles (Ene et al. 2005; Herbst et al. 2008). Previous studies show that land use near areas where turtles feed may influence disease rates, with elevated FP incidence grouped in watersheds with high nitrogen-footprints (Dailer et al. 2010; dos Santos et al. 2010; Van Houtan et al. 2010). Sites with high FP prevalence, such as the Manglar Bay sampling site in this study, may serve to amplify the disease transmission cycle, since tumors are a known source of viral transmission (Herbst et al. 1995; Herbst et al. 1996; Herbst et al. 2008). Future studies involving laser capture microdissection should include evaluation of a larger sample size to validate the accuracy of our conclusions. Determining exactly which circulating cells may be infected could have great value in further understanding of this disease and aid in devising more effective control measures.

## Methods

### Animals

In the Culebra archipelago of eastern Puerto Rico, two aggregations of green sea turtles were sampled for tumors over a period of three years. One aggregation was sampled in Manglar Bay, a basin on the island of Culebra which has relatively high levels of human activities and waste water runoff (18-18'13“ N, 065-15^′^19” W). The other aggregation was sampled at the more pristine cay preserve of Culebrita (18-18^′^53“ N, 065-13^′^44” W). Based on telemetry data tracking the movements of turtles found in Manglar Bay, these two aggregations were thought to commingle at night at a reef between the two sites (Diez et al. 2010). This is an assumption, however, since transmitters were not deployed on turtles found at Culebrita. According to mitochondrial DNA analysis, Manglar Bay and Culebrita green turtle aggregations recruit from multiple rookeries belonging to all five Atlantic and Caribbean Regional Management Units (Velez-Zuazo et al. 2010; Wallace et al. 2010).

A total of 126 green turtles were captured by netting and subsequently released, during six different time intervals over the three-year period 2004–2007. All were classified as juveniles, defined by a curved carapace length < 65 cm (Patricio et al. 2011). Because to date there is no available report on the somatic growth and maturity stages of these aggregations, this age-size classification is arbitrary; however it is based on similar, previously used classifications (Bresette et al. 2010; Chaloupka & Limpus 2001).

### Biopsy procedure

Following disinfection (Betadine) of the sampling tissue, tumors and/or normal skin from affected animals were either surgically removed or biopsied using a 6mm diameter punch. A punch biopsy of normal, non-tumored skin on the shoulder area was also taken from all animals, whether or not they were affected by FP. Tissues were placed in 10% buffered formalin for 12–24 hours, then cut in half and placed in diethyl pyrocarbonate (DEPC) treated phosphate-buffered saline (PBS, pH 7.6) and kept at 4°C. After transportation to the laboratory (1–2 weeks), they were kept at -80°C until assayed. All procedures were performed under the regulatory authorities of federal and state permit (US NMFS: Permit NO. 1253 and PRDRNA 12-EPE-04) and Animal Care and Use Protocols at the University of Georgia.

### PCR and sequencing protocol

Up to 25mg of tissue was minced into small pieces and placed into a 1.5ml microcentrifuge tube with 200μl of tissue lysis buffer containing 600mAU of Proteinase K.^d^ Tissues were incubated at 55°C until completely dissolved. Total DNA was extracted using the DNeasy tissue kit.^d^ Final concentration was determined by spectrophotometric analysis, using the ratios of absorption at 260nm versus 280nm to ensure DNA purity. A final concentration of 0.1-1 μg/μl was used for the PCR reactions. The oligonucleotide primers were designed according to the Hawaii green turtle herpesviral (fibropapilloma) genes, GenBank^a^ AF035003, representing a highly conserved region of the herpesviral DNA (Greenblatt et al. 2005; Quackenbush et al. 1998; Quackenbush et al. 2001). Three different pairs of primers were designed for three different genes of the herpesvirus – DNA polymerase catalytic subunit (UL30, *pol*), capsid maturation protease (UL26), and membrane glycoprotein B (UL27, *gB*). All primers were manufactured by Integrated DNA Technologies, Inc.^e^

The primary PCR primers and nested PCR primers were designed as follows: the forward and reverse primers for the primary PCR target to DNA polymerase catalytic subunit *pol* (5^′^ ---AGC ATC ATC CAG GCC CACA AT CTG--- 3^′^, 5^′^ ---CGG CCA GTT CCG GCG CGT CGA CCA--- 3^′^, respectively) result in an amplification product of approximately 445bp. These primers were used exactly as described by previous investigators (Lu et al. 2000). The forward and reverse primers for the nested PCR target to DNA polymerase catalytic subunit (5^′^ ---CGG CGA GCC GAA ACG CTC AAG G--- 3^′^, 5^′^ ---TCC GTT CCC CAG CGG GTG TGA A--- 3^′^) result in an amplification product of approximately 364bp.

The primary and nested PCR primers of capsid maturation protease were designed according to the 3357 to 5006 region of the Hawaiian green turtle herpesviral gene (GenBank^a^ AF035003), using Primer3 software^f^. The forward and reverse primers for the primary PCR target to capsid maturation protease (5^′^ ---AGA GCG AGG GTT TAG GCT GGA C--- 3^′^, 5^′^ ---CAA TGC CGC CCT TCC TCG TCG G--- 3^′^, respectively) result in an amplification product of approximately 495bp, and the forward and reverse primers for the nested PCR target to capsid maturation protease (5^′^ ---GAT CAC AAG GAC CGA TGC ACG G--- 3^′^, 5^′^ ---AGC GGT TTC ATC GTA TAT CGC G--- 3^′^, respectively) result in an amplification product of approximately 324bp.

The primary and nested PCR primers of membrane glycoprotein B were designed according to the 5155 to 7713 region of the Hawaiian green turtle herpesviral gene (GenBank^a^ AF035003), using Primer3 software^f^. The forward and reverse primers for the primary PCR target to virion membrane glycoprotein B (5^′^ ---GTG CGC ACT TCC GTA ATC TCG TCC--- 3^′^, 5^′^ ---CAG AGA CGC CAC CTT TAC TCA GGT--- 3^′^, respectively) result in an amplification product of approximately 534bp, and the forward and reverse primers for the nested PCR target to virion membrane glycoprotein B (5^′^ ---AGT AGG GAA GCA GCT CGT TGT G--- 3^′^, 5^′^ ---CGA CGT AAC GGT ATG GGA GCT G--- 3^′^, respectively) result in an amplification product of approximately 300bp.

To ensure that our findings were not the result of contamination, PCRs were run with two negative controls- both a chicken liver genome template and no genome template. The PCR products were electrophoresed to determine size, along with equivalent plasmid inserts for comparison. Prior to this study, the three gene targets were each cloned into plasmids, and the sequences were verified via multiple sequence alignment (Kang et al. 2008). These equivalent plasmid inserts were electrophoresed along with all test samples for size comparison, as positive controls. The PCR products with the same size and corresponding plasmid type were considered a positive product. The PCR products were resolved on 1% agarose gels. For three PCR products (one sample of each type of nested amplicon), bands of the appropriate size were excised and purified using the QIAquick Gel Extraction Kit.^d^ Capillary (Sanger) DNA sequencing was performed using the BigDye Terminator Kit^g^, and analyzed on a 3730 XL, 96-well capillary electrophoresis DNA sequencing system^g^ at the Georgia Genomics Facility at the University of Georgia. Viral sequences were compared to the Hawaiian green turtle herpesvirus, GenBank^a^ AF035003, using Clustal^b^ multiple sequence alignment to verify the gene targets according to DNA identity.

### Laser capture microdissection protocol

The biopsy sample evaluated was composed of non-tumored skin removed from the shoulder of a turtle without tumors. The tissue was placed in 10% buffered formalin for 1–2 weeks, then placed in a tissue cassette and embedded in paraffin. Paraffin-embedded tissue was cut into 5-10μm sections, three of which were mounted on a polyethylene naphthalate, 1.35μm membrane-slide. The tissue sections were stained using routine hematoxylin and eosin staining techniques. Epidermal and dermal tissues were microscopically visualized and differentiated, and four samples from each of the two tissue types were traced and microdissected. Microdissected samples were then cleanly removed using two separate isolation caps, one for each tissue type, which rested on a membrane and thus had no direct contact with the specimen.^h^ The membranes, contained within 1.5ml microcentrifuge tubes, were inundated with 180μl of tissue lysis buffer and 12mAU of Proteinase K.^d^ Tissues were successively incubated at 56°C and 90°C until completely dissolved. Total DNA was extracted using a DNA FFPE Tissue Kit,^d^ and primary and nested PCR for the polymerase gene target were performed, as previously described. The negative control of no genome template was used in these PCR assays; as a positive control, we used the DNA polymerase catalytic subunit *pol* equivalent plasmid insert as described above. The dermis and epidermis PCR products were resolved on a 1% agarose gel, and bands of the appropriate size were excised, purified using the QIAquick Gel Extraction Kit^d^, and sequenced^g^ by the Georgia Genomics Facility at the University of Georgia as previously described. These viral sequences were compared to that of the Hawaiian green turtle herpesvirus, GenBank^a^ AF035003, using Clustal^b^ multiple sequence alignment to verify the gene target according to DNA identity.

## Endnotes

^a^GenBank, National Center for Biotechnology Information, Bethesda, MD.

^b^Clustal W/X software, version 2.0.12, Conway Institute UCD, Dublin, Ireland.

^c^RNAlater, Qiagen, Valencia, CA USA.

^d^Qiagen, Valencia, CA USA.

^e^Integrated DNA Technologies (IDT), Coralville, IA USA.

^f^Primer3 software, version 0.4.0, Humana Press, Totowa, NJ USA.

^g^Applied Biosystems, Life Technologies Corporation, Carlsbad, CA USA.

^h^Molecular Machines and Industries, Haslett, MI USA.

## Abbreviations

CFPHV: Chelonid fibropapilloma-associated herpesvirus
PCR: Polymerase chain reaction
DNA: Deoxyribonucleic acid
FP: Fibropapillomatosis
RNA: Ribonucleic acid
DEPC: Diethyl pyrocarbonate
PBS: Phosphate buffered saline
US NMFS: UNITED STATES NATIONAL MARINE FISHERIES SERVICE.
